# Effects of the Occupational Ethics of Health Workers on Job Satisfaction—Focusing on Dental Technicians and Dental Hygienists

**DOI:** 10.3390/dj10090172

**Published:** 2022-09-14

**Authors:** Sun-Kyoung Lee, Jeong-Min Seong

**Affiliations:** 1 Department of Dental Technology, Kyungdong University, Wonju-si 26495, Gangwon-do, Korea; 2 Department of Dental Hygiene, Kangwon National University, Samcheok-si 25913, Gangwon-do, Korea

**Keywords:** dental hygienist, dental technician, ethics education, job satisfaction, professional ethics

## Abstract

This study investigates the effects of professional ethics on the job satisfaction of dental technicians and dental hygienists among health and medical personnel. From 1 July to 30 September 2021, a survey was conducted with 178 dental technicians and dental hygienists. Frequency analysis, correlation analysis, and multiple regression analysis were performed. The collected data were processed using SPSS version 22.0 statistical program (IBM, Armonk, NY, USA). The significance level was set to 0.05. As factors influencing the professional ethics of dental technicians and dental hygienists, diligence, reduced leisure, work orientation, and time saving were found to affect job satisfaction. In addition to morality, various elements of professional ethics and their causal relationships with job satisfaction were investigated. In the future, in preparation for the increasing ethical problems in the medical environment, education on professional ethics should be standardized and conducted more systematically.

## 1. Introduction

The ethical friction in everyday life due to the diversification and complexity of social structures can arise in a variety of settings, especially in the medical system that is related to human health, where ethical friction or judgment is required. These ethical problems cannot be solved with a simple and truthful approach, and rational decisions and judgments based on accurate ethical theories for a variety of situations become essential. This theory can be learned by the method of inquiry, called ethics.

If ethics has a mix of moral and legal tendencies, then professional ethics refers to the code of conduct required of those who perform a profession [1]. The knowledge and skills of professions are important in society, but can produce both benefit and harm. This is why a strict sense of professional ethics is required [2], especially in healthcare professionals who treat and care for patients at medical institutions.

For these professionals, personal ethics are of significance; personal ethics include traits such as diligence, honesty, and integrity, as well as community ethics such as service, responsibility, compliance, and workplace etiquette [3]. 

Dental technicians create, repair, and process teeth substitutes and orthodontic devices such as dentures in accordance with requests from dentists. Dental hygienists assist dentists in patient care and treatment and help people with proper oral hygiene [4]. 

Stricter codes of ethics are applied to dental technicians and dental hygienists compared to many other professions. In addition, the dental healthcare environment can involve a wide range of ethical issues including physical contact with patients, medical expenses, and privacy [5]. As such, trust and responsibility are expected between dental technicians/dental hygienists and patients. The ethical issues faced by these healthcare professionals become more complex as societal values and expectations change [6]. 

Schermerhorn (2000) defined job satisfaction as the level of positive or negative sentiment toward one’s job and the emotional response or attitude toward the physical and social situations in an organization [7]. According to Vecchio (2022), job satisfaction is an emotional response to one’s job and the personal experience related to job expectations [8]. 

Many researchers have addressed the relationship between professional ethics and job satisfaction. Saks (1996) reviewed previous studies on work ethics and work performance and found a strong correlation between professional ethics and job satisfaction [9]. Miller (2002) reported seven aspects of professional ethics that affect job satisfaction and organizational engagement: hard work, leisure, wasted time, morality, centrality of work, self-reliance, and delay of gratification [10]. Herzberg (1968) and Kim (2021) found that an individual’s professional ethics greatly affect his/her job satisfaction [11,12].

In the vein of that research, this study seeks to explore the effects of professional ethics on the job satisfaction of dental hygienists and dental technicians.

## 2. Materials and Methods

### 2.1. Participants

The questionnaire was distributed to 250 licensed dental technicians and dental hygienists across Korea, selected through convenience sampling, between 1 July and 30 September 2021. The questionnaire guaranteed confidentiality, as specified in the Personal Information Protection Act, and assured that the information in the questionnaire would not be used for any purpose other than research. The questionnaires were sealed with the respondents’ signatures or seal impressions. After excluding incomplete and non-returned responses, a total of 178 questionnaires were analyzed.

### 2.2. Research Design

This study used a version of the questionnaire tool developed by Petrovich [13], adapted and supplemented for the purpose of this study. The questionnaire consisted of questions on general participant information (sex, age, clinical experience, educational attainment, and religion), survey questions on professional ethics and education (ethical values, experiences in vocational ethics education, thoughts on vocational ethics education, necessity of vocational ethics education, and significance of ethical issues), and seven factors of the Protestant professional ethics (hard work, leisure, wasted time, morality, centrality of work, self-reliance, and delay of gratification) [10]. Each section consisted of five questions. Because the study subjects had no public risk and no studies were conducted on human derivatives, they did not obtain IRB approval.

The questions on professional ethics, ethical training, and job satisfaction offered five choices based on a Likert-type five-point scale. The survey was conducted as a self-report questionnaire.

Table 1 shows the reliability of the tools for measuring the respondents’ ethics.

### 2.3. Analysis Method

The collected data were analyzed with SPSS version 22.0 (IBM, Armonk, NY, USA), and the general characteristics, professional ethics, and education of the respondents were analyzed using descriptive statistics. 

The correlations between professional ethics factors and job satisfaction factors were analyzed using Pearson’s correlation coefficient. 

In addition, a multiple regression analysis was performed to understand the effects of professional ethics and ethics education on the job satisfaction of dental technicians and dental hygienists at a significance level of 0.05.

### 2.4. Definitions

Table 2 lists the definitions of the seven factors of the Protestant professional ethics.

## 3. Results

### 3.1. General Characteristics of the Respondents

The general characteristics of the respondents are summarized as follows: Of the dental technicians and dental hygienists, 59.6% were men and 40.4% were women. Regarding age, 57.9% of the respondents were in their 20′s, followed by respondents in their 30′s (19.1%), 40′s (11.2%), 50′s (10.7), and 60′s or older (1.1%). Clinical experience duration was 4 years or shorter in 46.6% of the respondents, followed by 5 to 9 years (15.2%), 10 to 14 years (14.0%), 15 to 19 years (9.0%), and 20 years or longer (15.2%). Graduates from three-year colleges comprised 55.1% of the respondents, four-year college graduates comprised 39.9%, and graduate school graduates comprised 5.1%. A religion was reported by 48.3% of the respondents, and the other 51.7% reported having no religion (Table 3).

### 3.2. Correlations between Factors of Professional Ethics and Job Satisfaction

Table 4 shows the correlation between the factors of professional ethics and job satisfaction. The relationship between professional ethics factors (hard work, leisure, wasted time, morality, centrality of work, and delay of gratification) and job satisfaction were positively correlated and statistically significant (*p* < 0.01). It could be seen that ethics about the profession were related to the satisfaction of the job.

### 3.3. Professional Ethics and Ethics Education of Dental Technicians and Dental Hygienists

Regarding their opinions on professional ethics and ethics education, 50.0% of the respondents reported that they are ‘very firm’ about their professional ethics, while 26.4% answered they are ‘sometimes confused’, and 23.6% chose ‘depending on the situation’. Experience in vocational ethics education was reported by 55.5%. As for the sufficiency of such education, 63.5% of the respondents chose ‘not complete’, 25.3% chose ‘sufficient’, and 11.2% chose ‘I do not know’. To the question regarding the necessity of vocational ethics education, 64.0% chose ‘need’, while 33.7% reported ‘I don’t know’, and 2.8% chose ‘not necessary’. When asked whether ethical issues will increase in the working environment in the future, 71.9% of the respondents answered ‘yes’, while 27.0% chose ‘I do not know’, and 1.1% chose ‘no’ (Table 5).

### 3.4. Effects of the Professional Ethics of Dental Technicians and Dental Hygienists on Job Satisfaction

Table 6 summarizes the effects of the professional ethics of dental technicians and dental hygienists on their job satisfaction. Among professional ethics factors, hard work (β = 0.334, *p* = 0.001), reduction in leisure (β = 0.098, *p* = 0.014), work centrality (β = 0.297, *p* = 0.001), and time saving (β = 0.109, *p* = 0.008) had positive effects on job satisfaction. The F-value, which signifies the fitness of the model, was statistically significant (*p* < 0.05). The corrected determination coefficient was 0.379, which means the variables explain 37.9% of the effect on job satisfaction.

## 4. Discussion

Healthcare professionals impact the quality of life of many people, be it positive or negative. If these professionals lack the ability to reasonably assert their professional beliefs, it will be difficult for them to advocate for healthcare users. Though not all issues are related to professional ethics, many are affected by morality. In order to understand the moral aspect of a situation, one should be mature enough to consider other views. In this study, 50% of the dental technicians and dental hygienists surveyed reported that they are ‘very firm’ about their professional ethics. However, 55% of the respondents reportedly did not receive any vocational ethics education, and 63.5% believed that their education was not sufficient. Many of the respondents (64.0%) felt that vocational ethics education is needed. Kim also reported lower scores for questions on vocational ethics education among occupational therapists, suggesting the need for vocational ethics education, thus, supporting the findings of this study [14]. Medical ethics are basically based on personal ethics, and it has been confirmed that there is a special code of ethics required by each person in the course of his or her professional career. Therefore, professional ethics can also be said to be an extension of personal ethics. The basic virtues of personal ethics, such as love, mercy, and the methodological ideology of the pursuit of common development and long-term mutual benefit, are the same. However, it can be seen as a separate virtue and norm required in the special situation of a profession as a more specialized branch of business, compared to the ethical relationship between friends, etc., which form a relationship centered on the basic values for human happiness.

In the context of the ethical judgments that may arise in the performance of the duties of dental technicians and dental hygienists, a clearer presentation of work ethics will be required. To orient this presentation, learners should focus on understanding and solving the problems of work ethics that they are encounter in their work lives and should be guided on the basis of examples that are closely related to the actual work situation. As for the correlations between professional ethics factors and job satisfaction, hard work, leisure, work centrality, morality, time saving, and delay of gratification were positively correlated. According to Jang et al. [15], the completion of vocational ethics education was reported by 34.8% of the students and 22.1% of the professors. These findings provide similar implications to the present study: healthcare professionals are aware of the seriousness of professional ethics issues but lack experience in actual ethics education.

The ethical friction in everyday life due to the diversification and complexity of social structures can occur in a variety of environments, especially in the medical system that is related to human health, where ethical friction or judgment is needed. In particular, in the medical environment, complex and diverse ethical issues such as physical contact with patients, exposure, costs, privacy, metastasis, reversal, and moral management of hospitals can emerge. These ethical problems cannot be solved with a simple and truthful approach, and rational decisions and judgments based on accurate ethical theories for a variety of situations become essential.

In recent years, the number of sexual harassment-related damages and complaints received by health care providers has been steadily increasing. Most cases involving sexual harassment stem from mistakes that result from a lack of ethical education or ongoing treatment practices, and there is a lack of recurrence prevention education for them. Therefore, it is believed that professional ethics must be preceded by the basic knowledge that one must possess as a professional through school education; through such schooling, it is possible to produce a health care practitioner who has cultivated ethical thinking by understanding the medical ethics that one should have in this role, and learning through professional ethics the basic duties of the job and the relationships with the patients involved, the human relationships, and the relationship with the profession.

Professional ethics factors that positively affect the job satisfaction of dental technicians and dental hygienists include hard work (β = 0.334, *p* = 0.001), reduction in leisure (β = 0.098, *p* = 0.014), work centrality (β = 0.297, *p* = 0.001), and time saving (β = 0.109, *p* = 0.008). As such, ideal, realistic, and proper vocational ethics education should consider these factors. A previous study reported that a healthcare professional with positive professional ethics likely will achieve better job performance and show higher engagement [16]. In addition, the awareness of professional ethics significantly affects job satisfaction [17]. These findings are consistent with those proposed in this study. 

Among the sectors of work ethic, hard work, wasted time, and morality have been shown to have a positive impact on organizational engagement. Ahn and Lee [18] have shown that professional ethics influences job satisfaction and organizational engagement. Cohen [19] and Witt [20] have shown that people with a high work ethic develop organizational engagement. Morrow [21] argues that work ethic influences organizational engagement. In addition, the correlation analysis shows that hard work, wasted time, and morality correlate with organizational engagement at a significance level of 0.01.

This study is limited in that it focuses on dental technicians and dental hygienists, who do not represent all healthcare professionals. This study needs to be followed by future research on a wider range of professionals, including the healthcare professionals not considered here.

## 5. Conclusions

In order to explore the effects of the professional ethics of dental technicians and dental hygienists on their job satisfaction, this study analyzed the answers of 178 respondents and found the following:

1. A positive correlation with job satisfaction was found for professional ethics factors of hard work, leisure, work centrality, morality, time saving, and delay of gratification.
2. The majority of the respondents were firm about their professional ethics and did not have sufficient vocational ethics education. The majority of respondents expected that professional ethics will take on greater importance in the future working environment and believe that additional vocational ethics education is needed.
3. Among the factors of professional ethics, hard work, reduction in leisure, work centrality, and time saving were found to significantly affect job satisfaction.

This study explored various factors of professional ethics other than morality and analyzed their correlations with job satisfaction. Professional ethics will be increasingly important in the work life of healthcare professionals experiencing rapid changes in the modern healthcare environment. As such, there is a need for systemic and continuous support by administrative and institutional efforts to provide vocational ethics education to professionals.

## Figures and Tables

**Table 1:** Measurement tool and reliability verification.

Occupational Ethics Factor	Number of Questions	Cronbach’s α
Hard work	5	0.914
Leisure	5	0.882
Wasted time	5	0.905
Morality	5	0.833
Centrality of work	5	0.820
Self-reliance	5	0.923
Delay of gratification	5	0.825

**Table 2:** Definitions of occupational ethics factors.

Occupational Ethics Factor	Definition
Hard work	A positive attitude toward job efforts
Leisure	One’s leisure activities and attitudes toward leisure time
Wasted time	Attitude toward and feeling rewarded by working
Morality	A norm that should be followed without harming others
Centrality of work	Effective use of time
Self-reliance	Overcoming difficulties with one’s efforts and judgment without relying on others and proper self-control
Delay of gratification	A willingness to postpone immediate satisfaction for a larger goal in the future

**Table 3:** General characteristics of the subjects.

Characteristics	Group	Dental Technician 106 (100%)	Dental Hygienist 72 (100%)	Total 178 (100%)
Sex	Male	68 (64.2)	4 (5.6)	72 (40.4)
	Female	38 (35.8)	68 (94.4)	106 (59.6)
Age (year)	20–29	55 (51.9)	48 (66.7)	103 (57.9)
	30–39	18 (17.0)	16 (22.2)	34 (19.1)
	40–49	15 (14.2)	5 (6.9)	20 (11.2)
	50–59	16 (15.1)	3 (4.2)	19 (10.7)
	≥60	2 (1.9)	0 (0.0)	2 (1.1)
Career duration	≥4	47 (43.3)	36 (50.0)	83 (46.6)
	5–9	10 (9.4)	17 (23.6)	27 (15.2)
	10–14	16 (15.1)	9 (12.5)	25 (14.0)
	15–19	12 (11.3)	4 (5.6)	16 (9.0)
	≥20	21 (19.8)	6 (8.3)	27 (15.2)
Education	College	58 (54.7)	40 (55.6)	98 (55.1)
	University	42 (39.6)	29 (40.3)	71 (39.9)
	≥Professional degree	6 (5.7)	3 (4.2)	9 (5.1)
Religion	Yes	56 (52.8)	30 (41.7)	86 (48.3)
	No	50 (47.2)	42 (58.3)	92 (51.7)

**Table 4:** Correlations between occupational ethics factors and job satisfaction.

	Job Satisfaction	Hard Work	Leisure	Wasted Time	Morality	Centrality of Work	Self Reliance	Delay of Gratification
Job satisfaction	1							
Hard work	0.577 **	1						
Leisure	0.440 **	0.257 **	1					
Wasted time	0.331 **	0.198 **	0.233 **	1				
Morality	0.301 **	0.415 **	0.021	0.101 *	1			
Centrality of work	0.225 **	0.278 **	0.099 *	0.161 **	0.333 **	1		
Self-reliance	0.002	0.189 **	0.095 *	0.054	0.107 *	0.244 **	1	
Delay of gratification	0.199 **	0.225 **	0.046	0.163 **	0.134 **	0.234 **	0.286 **	1

* *p* < 0.05, ** *p* < 0.01.

**Table 5:** Occupational ethics and education of dental technicians and dental hygienists.

Questions	Group	Dental Technician 106 (100%)	Dental Hygienist 72 (100%)	Total 178 (100%)
Work ethic	Very firm	64 (60.4)	25 (34.7)	89 (50.0)
	Sometimes confused	20 (18.9)	22 (30.6)	42 (23.6)
	Depending on the situation	22 (20.8)	25 (34.7)	47 (26.4)
Experience in vocational ethics education	Yes	41 (38.7)	36 (50.0)	97 (54.5)
	No	65 (61.3)	36 (50.5)	101 (55.5)
Thoughts on vocational ethics education	Sufficient	28 (26.4)	17 (23.6)	45 (25.3)
	Not complete	64 (60.4)	49 (68.1)	113 (63.5)
	I do not know	14 (19.2)	6 (8.3)	20 (11.2)
Necessity of vocational ethics education	Need	71 (67.0)	43 (59.7)	114 (64.0)
	It is not necessary	3 (2.9)	2 (2.8)	5 (2.8)
	I do not know	33 (31.1)	27 (37.5)	60 (33.7)
Opinions on whether ethical issues will grow in the working environment in the future	Yes	73 (68.9)	55 (76.4)	128 (71.9)
	No	1 (0.9)	1 (1.4)	2 (1.1)
	I do not know	32 (30.2)	16 (22.2)	48 (27.0)

**Table 6:** Effects of the professional ethics of dental technicians and dental hygienists on job satisfaction.

Factor	β	t	p	F	Adjusted R Square
Hard work	0.334	7.220	0.001	9.642	0.379
Reduction in leisure	0.098	2.474	0.014	9.642	0.379
Work centrality	0.297	7.254	0.001	9.642	0.379
Morality	0.035	0.843	0.400	9.642	0.379
Time saving	0.109	2.656	0.008	9.642	0.379
Independence	−0.062	−1.523	0.128	9.642	0.379
Self-reliance	−0.023	−0.558	0.577	9.642	0.379

## Data Availability

Not applicable.
